# Treatment with Oral ATP decreases alternating hemiplegia of childhood with *de novo ATP1A3* Mutation

**DOI:** 10.1186/s13023-016-0438-7

**Published:** 2016-05-04

**Authors:** Jun Ju, Shinichi Hirose, Xiu-Yu Shi, Atsushi Ishii, Lin-Yan Hu, Li-Ping Zou

**Affiliations:** Department of Pediatrics, Chinese PLA General Hospital, Beijing, 100853 China; Department of Pediatrics, School of Medicine, Fukuoka University, Fukuoka, Japan; Center of Epilepsy, Beijing Institute for Brain Disorders, Beijing, 100069 China; Department of Pediatrics, Beijing Chao-Yang Hospital, Chaoyang District, Beijing, 100020 China

**Keywords:** AHC, Epilepsy, Migraine

## Abstract

**Background:**

Alternating hemiplegia of childhood is an intractable neurological disorder characterized by recurrent episodes of alternating hemiplegia accompanied by other paroxysmal symptoms. Recent research has identified mutations in the *ATP1A3* gene as the underlying cause. Adenosine-5'-triphosphate has a vasodilatory effect, can enhance muscle strength and physical performance, and was hypothesized to improve the symptoms of paroxysmal hemiplegia.

**Methods:**

A 7-year-old boy with alternating hemiplegia of childhood who was positive for a de novo *ATP1A3* mutation was treated with adenosine- 5'- triphosphate supplementation orally as an innovative therapy for 2 years. Outcome was evaluated through the follow-up of improvement of hemiplegic episodes and psychomotor development. Side effects and safety were monitored in regularity.

**Results:**

With the dosage of adenosine-5'-triphosphate administration increased, the patient showed significantly less frequency and shorter duration of hemiplegic episodes. Treatment with adenosine-5'-triphosphate was correlated with a marked amelioration of alternating hemiplegia of childhood episodes, and psychomotor development has improved. The maximum dose of oral administration of adenosine-5'-triphosphate reached 25 mg/kg per day. Adenosine-5'-triphosphate therapy was well tolerated without complaint of discomfort and side effects.

**Conclusions:**

The 2-year follow-up outcome of adenosine-5'-triphosphate therapy for alternating hemiplegia of childhood was successful.

**Electronic supplementary material:**

The online version of this article (doi:10.1186/s13023-016-0438-7) contains supplementary material, which is available to authorized users.

## Background

Alternating hemiplegia of childhood (AHC, MIM 104290) is a rare and intractable neurological disorder with an early manifestation and poor prognosis. The characteristic clinical symptom of AHC is recurrent alternating episodes of hemiplegia that last from minutes to days, and this is accompanied by other paroxysmal symptoms including dystonia, nystagmus, autonomic disturbances, and seizures, as well as progressive cognitive and motor impairment [1, 2]. The diagnosis of AHC was previously based on clinical manifestations, and the most widely accepted clinical criteria to diagnose AHC were proposed by Bourgeois et al. In 2012, researchers identified mutations in the *ATP1A3* gene (MIM 182350) encoding the sodium-potassium (Na^+^/K^+^) ATPase α_3_ subunit as the primary genetic cause of sporadic AHC [3–5].

Adenosine-5'-triphosphate (ATP) is a purine nucleotide found in every human cell, and its most important function is to transfer energy. Recent studies reported that extracellular ATP has a vasodilatory effect and is capable of enhancing physical performance [6]. Therefore, we hypothesized that ATP might ameliorate motor dysfunction in hemiplegic limbs and have a therapeutic effect in patients with AHC.

Here we report a patient with AHC and a *de novo* splice-site mutation in *ATP1A3* (c.2542 + 1 G > A) who was treated with oral ATP, which decreased the frequency and severity of hemiplegic episodes.

### Method

#### Clinical course and diagnosis

The patient was a 7-year-old boy who first presented with recurrent hemiplegic episodes at the age of two and a half. The extent of hemiplegia varied and he usually experienced right-sided hemiplegia with an unsteady gait and was prone to falling. Occasionally, he had bilateral involvement with flaccid tetraparesis. The hemiplegic episodes lasted several minutes and ranged from once a week to more than 10 times per day. The first hemiplegic episode occurred when he was recovering from a respiratory tract infection; in the following days, the parents noted that triggering factors included hunger, fear, fatigue, cold, agitation, and emotional stress.

This patient was born to non-consanguineous parents after an uneventful pregnancy. There was no family history of migraine, epilepsy, or Parkinson’s disease. Neurological examinations were unremarkable when the patient was not experiencing hemiplegic episodes. His early speech and motor development were largely normal; he was able to say individual words at the age of 16 months and walked unaided at 18 months old. The patient was clumsy and lacked fine motor in coordination. He had poor cognitive processing compared with normal children his age. During the initial course of the disease, the patient was diagnosed with epilepsy accompanied with Todd’s paresis in the local hospital and treated with oxcarbazepine (0.3 g, Q12h, po), which had no effect on the hemiplegic episodes.

The patient was first seen at the Department of Pediatrics in Chinese PLA General Hospital at age of 3.5 years. He constantly experienced hemiplegic episodes that generally lasted 10–20 min, with the longest lasting about 40 min after a long journey. At their most frequent, the patient experienced more than 10 attacks per day, and this could continue for up to 2 weeks. Muscle strength of affected limb was grade 3, and muscle tension decreased, which could not recover during interracial period because of constant episodes. The patient exhibited no pathologic reflex. Occasionally, the patient experienced external strabismus affecting his left eye during the attacks. This patient did not present with dystonic spells, nystagmus, seizure, or autonomic disturbance (Additional file 1: Video 1: The patients’ parents provided written informed consent for releasing the video of the patient as academic publication material). Video-electroencephalography (EEG) revealed no simultaneous changes during the hemiplegic spells. There were no metabolic abnormalities; lactic acid/pyruvic acid exercise tests were unremarkable; and EEG, magnetic resonance imaging, and magnetic resonance angiography of the brain were normal. Based on the clinical manifestation and work-up results, a diagnosis of AHC was considered. No tandem mass spectrometry was performed and no mitochondrial and/or genetic testing was done. Oxcarbazepine was stopped, and flunarizine (5 mg/day) was administered for 1 year, but had no obvious effect on attack frequency or duration, therefore was stopped. Coenzyme Q10 (10 mg, Q12h, po) and carnitine (1 g, once a day) were then prescribed together, but no beneficial effects were observed. Carnation was stopped because of its expensive.

**Fig. 1:**
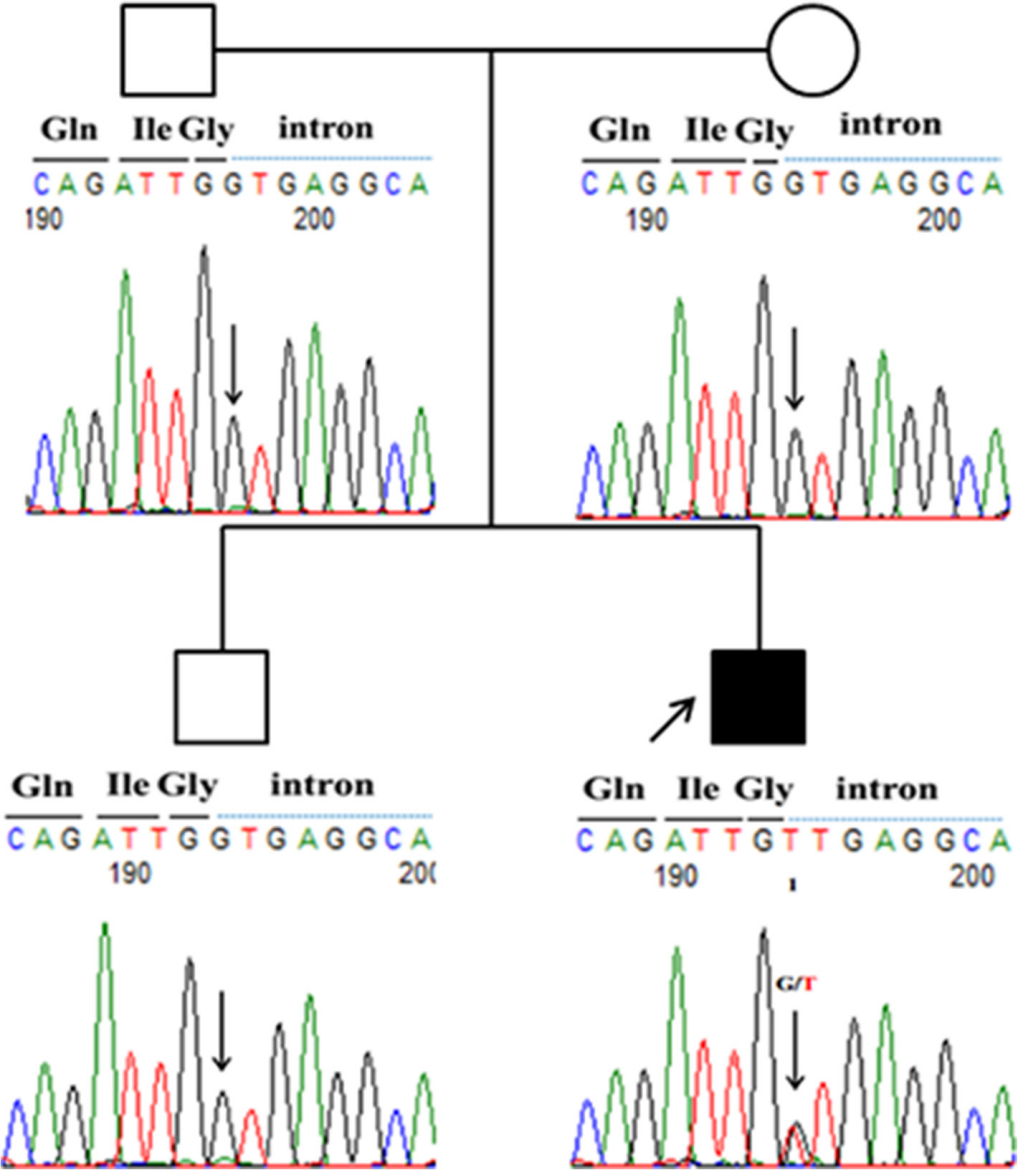
Chromatograms of the *de novo ATP1A3* mutation. Data were obtained by Sanger sequencing during the confirmation process. The *black square* represents the proband. *Black solid lines* indicate exonic nucleotide sequences, and *blue dotted lines* indicate intronic nucleotide sequences. A mutation (c.2542 + 1 G > A) in intron 18 was identified in this patient

When the patient was 5 years and 10 months, a genetic diagnosis of AHC due to *ATP1A3* mutation was confirmed. Specifically, the patient was determined to possess a *de novo* splice-site mutation (c.2542 + 1 G > A) in intone 18 of the *ATP1A3* gene that was not present in his parents or elder brother (Fig. 1).

#### ATP treatment

This research was approved by the ethics committees of Chinese PLA General Hospital, and the patient’s parents provided informed consent. Adenosine triphosphate disodium reagent was produced by Guangzhou Baiyunshan Guanghua Pharmaceutical Co. Ltd. At age of 6 years, ATP was orally administered at an initial dosage of 2 mg/day.d. When the hemiplegic episodes were aggravated, ATP was intravenously administered at 40 mg/day for 10 days. His parents were asked to record paroxysmal symptoms and side effects in detail, and blood counts and liver and renal function were regularly monitored every 3 months. His psychomotor development was also clinically assessed. Because of the worry from parents, he also continued taking coenzyme Q10 (10 mg twice a day) in the first year of treatment.

## Results

The ATP and coenzyme Q10 dosages were 20 mg twice a day (2 mg/kg/day), and 10 mg twice a day, respectively. The patient experienced fewer hemiplegic attacks that were shorter than those experienced before initiating ATP. When hemiplegic spells were evoked by a respiratory tract infection or fatigue, the symptoms would remit 2–3 days after intravenous ATP administration.

The dosage of ATP was gradually increased to 100 mg three times a day (15 mg/kg/day), and the patient experienced a marked reduction in the number of attacks, including a month without an event. Intravenous ATP administration was no longer needed even if physical or emotional triggers were present.

**Fig. 2:**
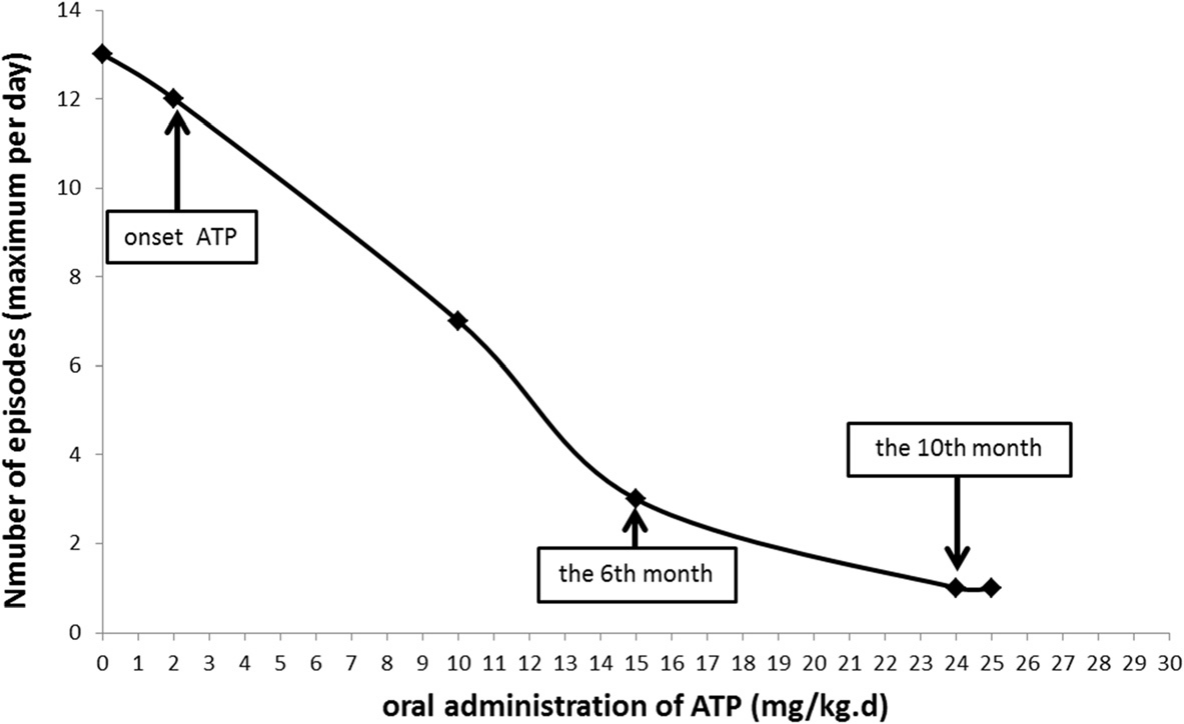
Daily frequency of alternating hemiplegia episodes at different ATP dosages. The numbers of hemiplegic episodes at different ATP administration are noted. As the ATP dosage increased, the patient experienced markedly fewer hemiplegic episodes

When titrating the dosage of ATP to 160 mg three times a day (24 mg/kg/day), the patient got mild hemiplegia of the right limbs lasted for several minutes only three times over 2 months, and the paroxysms were consistently provoked by crying and overtiredness. Despite this, the episodes were much less severe (Fig. 2). The neurological physical examination shows muscle strength of affected limb was grade 4, and muscle tension decreased in the stage of attack, while no abnormal physical signs were observed in interphase.

After the patient’s hemiplegic episodes were markedly reduced, the patient showed noticeable improvements in balance and fine motor coordination. He was able to run without falling over (Additional file 2: Video 2), and his academic performance improved including basic mathematical skills such as addition and subtraction and reciting nursery rhymes. Despite these improvements, his memory and perception abilities were delayed.

With the satisfactory result accompanied of titrating the dosage of ATP, the parent was tried to discontinue coenzyme Q10 after treatment for 1 year. As a result, there was no significant change in the frequency and severity of attacks after ATP monotherapy.

At present, we have followed up for 2 years. The maximum dose of oral ATP alone has increased to 240 mg three times a day (25 mg/kg/day). There was still a hemiplegic episode every several days. We did not increase the dosage based on the acceptable effects from parents. Most particularly, every dose increase acquired attack-free for about ten days.

After administered with ATP orally for 2 years, the patient was tolerated well with no nausea, vomiting, abdominal pain, or gastrointestinal discomfort. No complaints of adverse events happened. The patient’s liver parameters (glutamic-pyruvic transaminase, glutamic-oxalacetic transaminase), kidney parameters (including urea, creatinine, uric acid), blood albumin, bilirubin, electrolytes (sodium, potassium, calcium, magnesium) were monitored every half a year and within normal limits, except serum uric acid occasionally reached slightly higher than the upper limit, which indicated that prolonged ATP administration was safe.

## Discussion

Since AHC was first described, various drugs have been administered to reduce symptoms, but none has been particularly effective. Flunarizine is the first-line treatment, and several studies have reported that it reduces the severity, duration, and frequency of hemiplegic spells [7]. However; it has proven ineffective in a considerable number of patients, underscoring the need for effective therapies to treat AHC.

Here we report the case of AHC in a patient who had a previously reported [4, 8] *de novo* splice-site mutation in *ATP1A3* (c.2542 + 1 G > A). This mutation was rare in previous report and detected in only one patient with intellectual disability, walking problem, dystonia, hemiplegic attack, and epilepsy. The patient in our study presented a similar phenotype except epilepsy. The larger number of patient with same genotype were needed to define the clinical profiles.

In this case, we found patient showed a dramatic reduction in hemiplegic episodes and noticeable improvements in motor ability with initiation of oral ATP therapy. This treatment was well tolerated with no adverse effects in 2 years. There were notable reductions in the frequency, duration, and extent of hemiplegic episodes. A temporal correlation between treatment and therapeutic effects was observed; moreover, the patient’s symptoms seemed to improve in a dose-dependent fashion. This case suggests that ATP might be an effective medication for AHC treatment.

The *ATP1A3* gene encodes the α_3_-subunit of the sodium-potassium (Na^+^/K^+^) ATPase, which is well known as transmembrane ion pumps generating chemical and electrical gradients of Na^+^ and K^+^ across the membrane. The gradients have a key role in cellular physiological functions, including electrical excitability of nerves and muscles. The *ATP1A3* mutations would result in abnormal protein expression of Na^+^/K^+^ ATPase. Moreover, ATP, serving as a direct source of energy in living cell, provides energy for the ATPase. Supplementation of ATP may improve the dysfunction of Na^+^/K^+^ ATPase to alleviate symptoms. However, functional studies of ATPase and genotype-phenotype correlations of *ATP1A3* mutations should be further investigated [8].

Extracellular ATP can influence neurotransmission, muscle contraction, cardiac function, platelet function, vasodilatation, muscle oxygenation, and liver glycogen metabolism. ATP has been used as an adjuvant therapy for progressive muscle atrophy, supraventricular tachycardia, cerebral hemorrhage, cardiac insufficiency, myocardial diseases, paroxysmal atrial tachycardia, and hepatitis [6]. A recent randomized, double-blind, controlled study confirmed that oral supplementation with ATP (120 mg three times a day) increased quadriceps strength and benefitted patients recovering from total knee arthroplasty [9]. Another group reported that oral ATP administered as a disodium salt increased blood flow in exercising animals and humans, which could improve oxygen and nutrient delivery to the muscle and accelerate the removal of metabolic waste products such as lactate and urea. [10] Therefore, ATP might increase muscle strength in hemiplegic limbs via vasodilatation and increased blood flow.

There are limited data describing ATP dosages, especially in pediatric patients. Oral ATP at a maximum daily dose of 5000 mg was given to healthy adults for 28 days, and the findings of that study suggested that chronic ATP administration is safe with regard to liver and kidney parameters [11]. In this case, the maximum dosage was 480 mg/day, and the patient did not complain of discomfort and manifested no complications.

There are few studies regarding therapy for AHC. Although we only treated a single case, this patient showed a significant response to ATP, indicating that its use in AHC is worth exploring. Further large-scale studies involving patients with confirmed AHC are needed to definitively determine optimum dosing regimens and confirm its efficacy.

## Conclusions

The 1-year follow-up outcome of adenosine-5'-triphosphate therapy for alternating hemiplegia of childhood was successful.

## Additional files

Additional file 1: Video 1. This video was recorded in June 2011, at that time the patient was at age of 3.5 years and first seen at the Department of Pediatrics in Chinese PLA General Hospital. He frequently experienced right-sided hemiplegia with an unsteady gait and was prone to falling. (MOV 18913 kb) Additional file 2: Video 2. This video was recorded in December 2014. After treatment of ATP, this patient’s hemiplegic episodes were markedly reduced, the patient showed noticeable improvements in balance and fine motor coordination. (MOV 71448 kb)

## Abbreviation

AHC: alternating hemiplegia of childhood
ATP: adenosine-5'-triphosphate
EEG: electroencephalography
